# MACC1 Contributes to the Development of Osteosarcoma Through Regulation of the HGF/c-Met Pathway and Microtubule Stability

**DOI:** 10.3389/fcell.2020.00825

**Published:** 2020-12-23

**Authors:** Jia Wen, Yi Xie, Yingqiang Zhang, Jiazhen Li, Jiaping Li, Yan Zhang, Xinchang Lu, Yi Zhang, Yongkui Liu, Tao Liu, Longqing Li

**Affiliations:** ^1^Department of Orthopedics, The First Affiliated Hospital, Zhengzhou University, Zhengzhou, China; ^2^Department of Neurology, The First Affiliated Hospital, Zhengzhou University, Zhengzhou, China; ^3^Department of Interventional Oncology, The First Affiliated Hospital, Sun Yat-sen University, Guangzhou, China; ^4^Department of Interventional Radiology, The Seventh Affiliated Hospital, Sun Yat-sen University, Guangzhou, China

**Keywords:** MACC1, osteosarcoma, HGF/c-Met, proliferation, angiogenesis, microtubule stability

## Abstract

Osteosarcoma (OS) is the most prevalent human bone malignancy, and presents a global annual morbidity of approximately five cases per million. Notably, precise and efficient targeted therapy has become the most promising strategy for the treatment of OS; however, there is still an urgent need for the identification of suitable therapeutic targets. Metastasis-associated in colon cancer 1 (MACC1) was first identified in colon tumors by differential display RT-PCR, and was shown to be involved in the regulation of colon tumor growth and metastasis through the hepatocyte growth factor (HGF)/c-Met signaling pathway. Additionally, MACC1 overexpression has been reported to induce the growth of several types of cancers, including glioblastoma multiforme and gastric cancer. However, whether MACC1 also plays a role in the progression of OS remains unclear. In this study, we found that MACC1 was highly expressed in human OS tissues, as well as in U-2OS and MG-63 cells, when compared with normal tissues and osteoblasts, respectively. Our data further indicated that MACC1 expression was correlated with several clinicopathological features of OS. Through *in vitro* assays, we found that MACC1 depletion markedly suppressed the proliferative ability of both OS cells and endothelial cells, and inhibited the angiogenic capacity of endothelial cells. Similarly, MACC1 depletion inhibited tumor growth, metastasis, and angiogenesis in mice. Mechanistically, we found that MACC1 could bind to the *MET* promoter, and enhanced the proliferation of both OS cells and endothelial cells through the HGF/c-Met signaling pathway. Furthermore, we show that MACC1 also promoted angiogenesis by regulating microtubule dynamics, thereby promoting the progression of OS. Our results indicate that MACC1 may be a new and promising therapeutic target for the treatment of OS.

## Introduction

Osteosarcoma (OS) is the most frequently occurring primary bone malignancy in children and young adults, and accounts for 20–35% of all bone tumors (Raimondi et al., 2017; Liu et al., 2018). At present, the standard therapy for OS patients is a combination of preoperative chemotherapy, surgical resection, and adjuvant postoperative chemotherapy (ElKordy et al., 2018). Although considerable improvements have been made in surgical resection and combined therapeutic strategies, OS patients with lung metastasis and advanced clinical stage, the leading causes of OS-related death, have poor survival rates (Cong et al., 2018). Therefore, to improve the prognosis, new biomarkers and/or therapeutic targets must be identified for the treatment of patients with OS.

Metastasis-associated in colon cancer 1 (MACC1), first identified in 2009 as a differentially expressed gene in human colon cancer tissues, has been identified as a critical regulator and biomarker for cancer progression and metastasis in over 20 types of cancers, including bladder, colon, and esophageal cancers (Arlt and Stein, 2009; Qiu et al., 2011; Ashktorab et al., 2016; Qian et al., 2017). Several studies have indicated that MACC1 has potential as a prognostic marker for cancer progression. Importantly, high levels of circulating MAAC1 protein can predict the increased risk of metastasis formation in cancer (Hagemann et al., 2019; Qi et al., 2019). Moreover, MACC1 expression was shown to correlate with the survival of patients with pancreatic, breast, and lung cancers. Elevated MACC1 expression has also been associated with treatment response and resistance to chemotherapeutic agents (Muendlein et al., 2014; Sueta et al., 2015; Guo et al., 2018; Qi et al., 2019). Although these observations implicate MACC1 as a critical regulator of cancer progression, whether MACC1 also has a potential role in the development and progression of OS remains unclear.

Importantly, MACC1 is also indicated to be a transcriptional regulator of c-Met which is receptor tyrosine kinase aberrantly activated in human cancers. Mechanistically, MACC1 may contribute to cancer progression *via* the MACC1/hepatocyte growth factor (HGF)/c-Met axis (Boardman, 2009), as has been reported for ovarian cancer, gastric cancer, and HCC (Chang et al., 2012; Li et al., 2015; Dong et al., 2018). Furthermore, MACC1 was shown to enhance the migratory, invasive, and angiogenic potential of gastric cancer through the phosphorylation of c-Met and AKT, which stimulates the expression of TWIST1/2, epithelial–mesenchymal transition-associated transcription factors (Zhu et al., 2018). In addition, it is known that depletion of MACC1 also leads to disruption of the HGF/c-Met signaling pathway and sensitizes chemotherapy-resistant cancer cells (Sueta et al., 2015).

Angiogenesis is a biological process that results in the sprouting of new vessels from preexisting ones, and is a key event in the metastasis of solid tumors. Angiogenesis is achieved through the migration and proliferation of endothelial cells, a process that requires rapid and dynamic microtubule organization. Inappropriate angiogenesis often facilitates cancer cell metastasis. Several studies have also indicated that MACC1 influences tumor angiogenesis *via* a series of complicated pathways (Horvat et al., 2017; Peng et al., 2019). Interestingly, the ZU5-UPA-DD domain present in the MACC1 protein is known to function in the regulation of cytoskeletal dynamics, suggesting that MACC1 may also influence microtubule-mediated processes, such as cell migration and angiogenesis (Kokoszynska et al., 2009).

In this study, we found that MACC1 was highly expressed in human OS tissues, and the expression of MACC1 was correlated with the prognosis and several clinicopathological features of OS patients. Mechanistically, we show that MACC1 modulated the proliferation of OS cells *via* the MACC1/HGF/c-Met axis, both *in vitro* and *in vivo*. Furthermore, we also show that MACC1 depletion suppressed angiogenesis in a microtubule-dependent manner. Our results confirmed that MACC1 is involved in OS progression, and revealed the associated regulatory mechanisms, which suggest that MACC1 may be a promising therapeutic target for the treatment of OS.

## Materials and Methods

### Clinical Samples

This retrospective study was performed at the First Affiliated Hospital of Zhengzhou University and the First Affiliated Hospital of Sun Yet-sen University. A total of 223 patients, diagnosed with OS by histopathologic examination, were enrolled from January 2011 to December 2018. Biopsy samples and medical records were included in the study. The clinicopathological characteristics of OS patients are displayed in Table 1. Tumor volumes were measured *via* magnetic resonance imaging and calculated according to the following formula: V = long axis × short axis2 × π/6. Disease-free survival was defined as the time from the day of radical excision to the day of recurrence or metastasis, death from any cause, or the most recent follow-up. Overall survival was defined as the time between the day of diagnosis and the day of death from any cause or the most recent follow-up.

**TABLE 1:** Clinicopathological characteristics of osteosarcoma patients.

Characteristics		Cases	%
Gender	Male	145	65
	Female	78	35
Age (years)	< 15	54	24.2
	15–20	67	30
	21–30	34	15.2
	31–40	21	9.4
	> 40	47	21.1
Primary site	Limbs	175	78.5
	Others	48	21.5
Tumor staging	I	13	5.8
	II	189	84.8
	III	21	9.4
Tumor volume	<200 cm^2^	131	58.7
	>200 cm^2^	92	41.3
Radical excision	Yes	197	88.3
	No	26	11.7
Recurrence/metastasis	Yes	110	55.8
	No	87	44.2
Outcome	Alive	114	51.1
	Lost to follow-up	36	16.1
	Death	73	32.7

### Immunohistochemistry

Expression of MACC1, HGF, and c-Met in human OS tissues and adjacent noncancerous tissues was detected by immunohistochemistry. Excised human tissues were fixed in 4% formaldehyde at room temperature, embedded in paraffin, and sliced into 4-μm sections. Antigen retrieval was performed in citrate buffer (pH 6.0). Slides were subsequently blocked with goat serum (OriGene Technologies, Inc., Beijing, China) at room temperature for 15 min, followed by incubation with primary antibodies targeting MACC1 (rabbit polyclonal; ab106579, 1:500; Abcam, Cambridge, MA, United States), Ki-67 (ab15580, 1:1000; Abcam), Proliferating Cell Nuclear Antigen (PCNA) (ab92552, 1:500; Abcam), HGF (rabbit polyclonal; ab83760, 1:100; Abcam), and c-Met (rabbit monoclonal; ab51067, 1:250; Abcam) at 4°C overnight. After washing three times with PBS, the sections were incubated with biotinylated secondary antibodies for 1 h at room temperature, and visualized using diaminobenzidine as a chromogen substrate.

All images were obtained with an Olympus CX31 (Olympus Corporation, Tokyo, Japan). The quantification criteria were divided into four groups, comprising the following scores: no staining = 0, weak staining = 1, moderate staining = 2, and strong staining = 3. For percentage of stained cells, the following criteria were used: no staining = 0, 1–10% of cells stained = 1, 11–50% = 2, 51–80% = 3, and 81–100% = 4. In this study, scores 0–3 were assigned to low expression levels, and 3–6 to high expression levels, as determined by multiplying the percentage of positive cell percentage by the staining intensity. Immunostaining was manually assessed by Kaplan–Meier survival analysis.

### Cells and Transfection

U-2OS, MG-63, Saos-2, HOS, and HCO cell lines were maintained in our lab, while established human umbilical vein endothelial cell line (HUVEC) was obtained from ATCC, and maintained in DMEM and RPMI-1640 medium, respectively, supplemented with 10% fetal bovine serum (FBS) at 37°C in a 5% CO_2_ incubator.

Metastasis-associated in colon cancer 1-targeting shRNA plasmids or pcDNA3.1-MACC1 plasmids were transfected into OS cells using lipofectamine 2000 (11668019, Invitrogen, Carlsbad, CA, United States). MACC1 stable knockout U-2OS cells and HUVECs were generated by lentiviral infection. The rescue experiment was the method that transfect the plasmids one by one. At 0 h, cells were transfected with control or MACC1 shRNA plasmids. After 24 h, MACC1 was depleted, and the vector or MACC1 overexpression plasmids were transfected into the OS cells or HUVECs to rescue its expression. Another 24 h later, the in vitro cell assays were performed.

The shRNA sequences for MACC1 were as follows: for shRNA1, F: AGGUAAGAUUGGACUUGUAtt and R: UAC AAGUCCAAUCUUACCUct; and for shRNA2, F: AGUUA GUACGACUCACAAAtt and R: UUUGUGAGUCGUACUA ACUtt.

Cell cultures were treated with HGF (Sigma, St. Louis, MO, United States) at 20 ng/ml for 24 h.

### Immunoblot Assays

Total protein was extracted from cells and tissues using lysis buffer (60 mM Tris-HCl, pH6.8, 2% SDS, 20% glycerol, 0.25% bromophenol blue, 1.25% 2-mercaptoethanol and protease inhibitor cocktail). The protein concentration was measured by the bicinchoninic acid assay (Beyotime Institute of Biotechnology, Shanghai, China) according to the manufacturer’s instructions. The protein samples (30 μg/lane) were separated by SDS–PAGE and transferred onto polyvinylidene fluoride membranes. After blocking with 5% BSA in TBST containing 0.1% Triton X-100 in TBS, the membranes were incubated with primary antibodies against MACC1, HGF, and beta-actin (mouse monoclonal; TA-09, 1:4,000; ZSGB-Bio, Beijing, China) at 4°C overnight. After washing, the PVDF membranes were incubated with the corresponding secondary antibodies for 2 h at room temperature. Staining was detected using an ECL kit and analyzed with ImageJ (1.6.024; National Institutes of Health, Bethesda, MD, United States).

### Colony Formation Assays

Osteosarcoma cells with or without MACC1 ablation were seeded at approximately 1 × 10^3^ cells/well into a 6-well culture plate. After incubating for 14 days, adherent cells were fixed in PFA, and then stained with 0.2% crystal violet for 30 min. The colonies were then imaged and the colony numbers manually counted.

### CCK-8 and MTT Assays

Cells transfected with control or MACC1-targeting shRNA plasmids were seeded into 96-well plates at 2 × 10^4^ cells/well and maintained for 48 h. The cells were then treated with CCK-8 solutions according to the manufacturer’s instructions (Dojindo Molecular Technologies, Rockville, MD, United States). The absorbance at 450 nm of each well was assessed by microplate reader.

For MTT assays, the cells were incubated with MTT for 4 h and then washed twice with PBS. The OD_570_ was measured with a microplate reader for evaluation of cell viability.

### Tube Formation Assay

Human umbilical vein endothelial cell lines were plated into a 24-well plate precoated with Matrigel (1:1 dilution with RPMI-1640 medium). Images were obtained at the indicated times with a fluorescence microscope (Carl Zeiss, Jena, Germany), and the degree of tube formation was quantified by measuring the total tube length and number of nodes.

### Wound Healing Assays

Human umbilical vein endothelial cell lines were transfected with control or MACC1-targeting shRNA plasmids and grown for 48 h. The cells were then wounded by scraping with a 200-μl pipette tip, followed by washing. Subsequently, complete culture medium was added to induce wound healing. Images were taken at 0 and 24 h to evaluate the extent of cancer cell migration.

### Transwell Assays

Human umbilical vein endothelial cell lines transfected with or without MACC1 knockdown were seeded into the upper chamber of Transwells Matrigel-coated (20%; BD, Biosciences, San Jose, CA, United States) chambers with 8-μm pore-size membranes in serum-free DMEM and incubated at 37°C for 30 min. Then, the upper cells were induced to migrate toward the bottom chambers containing DMEM supplemented with FBS. After incubation for 48 h, cells in the upper chamber were removed with a cotton swab, and the migrated cells were fixed in 4% PFA, stained with 0.2% crystal violet (Sigma–Aldrich; Merck KGaA, Darmstadt, Germany), and quantified.

### RNA Extraction and Quantitative PCR

Total RNA was isolated using Trizol (15596026, Invitrogen) according to the manufacturer’s instructions. Genomic DNA was removed with DNase. And mRNA was reverse-transcribed into cDNA using the PrimeScript^TM^ RT Reagent kit (RR047A; Takara Bio, Inc., Otsu, Japan). Quantitative PCR was performed using the SYBR Ex Taq kit (638319, Takara Bio, Inc.) on a 7900HT Fast Real-Time PCR system (Thermo Fisher Scientific, Waltham, MA, USA). Beta-actin was used as an internal control. The sequences of the primer pairs used in this study are as follows: MACC1, F: 5′-TGGACATTTTAGACGACACAGC-3′, R: 5′-CCTCCTTGATGGTTTACTTTGC-3′; c-Met: F: 5′-GAGAAG CCCAAGCCCATCC-3′, R: 5′-GCCCAGGGCTCAGAGCTT-3′; HGF: F: 5′-GAATGACACTGATGTTCCTTTGG-3′, R: 5′-GGA TACTGAGAATCCCAACGC-3′; GAPDH: F: 5′-GCACCGTCA AGGCTGAGAAC-3′, R: 5′-ATGGTGGTGAAGACGCCAGT-3′.

### Tumor Growth Assays

All animal experiments in this study and approved by the Ethics Committee of our hospital. Male nude BalB/c mice (*n* = 60, 7–8 week) were bought from Beijing Vital River Laboratory Animal Technology Co., Ltd. (Beijing, China) and maintained in a SPF room, with a 12h light/12 h dark cycle at a temperature of 18–23°C and with food and water *ad libitum*. Cells transfected with control or MACC1 shRNA lentiviral constructs were subcutaneously implanted into nude mice to induce tumors. After 2 weeks, the mice were euthanized and the tumors were excised every 3 days. The volume of each tumor was measured. After 29 days, all the tumors were isolated from the mice and imaged.

### Immunostaining

To promote microtubule depolymerization, cells were plated on ice for 5 and 15 min. Cells were fixed in 2% paraformaldehyde for 20 min and permeabilized with 1% Triton X-100 in PBS (PBST) for 10 min. The cells were then blocked with 2% BSA in PBST and incubated with antibodies against β-tubulin (ab179513, 1:500, Abcam) and γ-tubulin (ab11316, 1:500, Abcam) at 4°C overnight. Immune complexes were stained with Alexa 488- or Alexa 546-conjugated secondary antibodies (Invitrogen). Nuclei were counter-stained with DAPI (Sigma–Aldrich). Each experimental group comprised 6 wells, and five fields were imaged per well.

### Chromatin Immunoprecipitation

Cells were seeded and allowed to attach. And then cells were transfected with pcDNA3.1-MACC1 and control pcDNA3.1 plasmid as described above. Subsequently, cells were crosslinked with 1% formaldehyde (Sigma-Aldrich Tokyo, Japan) and quenched in 0.125 M glycine. Sheared chromatin via a Bioruptor^®^ (Cosmo Bio Co., Ltd., Tokyo, Japan), and DNA and protein complex was pulled down by MACC1 antibody for 2 h at room temperature. Isolated DNA was further purified using a QIAquick PCR Purification kit (Qiagen) and amplified using AmpliTaq Gold^®^ 360 Master Mix (Life Technologies Japan). The primer sequences of c-Met promoter are F: 5′-CTAACTTCAGACTGCCTGAGC-3′, R: 5′-CACCACCCAGAGGGAAATC-3′.

### Statistics

All data were analyzed with GraphPad v.6.0 (GraphPad Software, La Jolla, CA, United States). All data in this study are representative of at least three independent experiments, and are expressed as means ± SEM. Additionally, χ^2^ analysis was performed to assess the correlation between clinicopathological features and MACC1 expression. Kaplan–Meyer analysis was performed to evaluate prognosis. The Student’s *t*-test was used for comparisons. ^∗^ Indicates *p*<0.05, ^∗∗^indicates *p* < 0.01, and ^∗∗∗^ indicates *p* < 0.001.

## Results

### MACC1 Was Highly Expressed in Human OS Tissues and Correlated With Prognosis and Clinicopathological Characteristics

**FIGURE 1:**
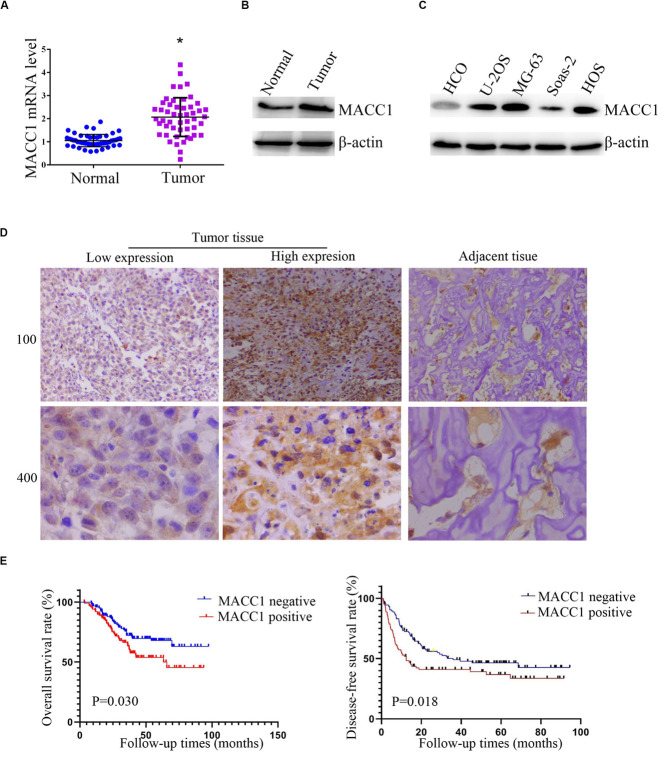
MACC1 is highly expressed in human osteosarcoma (OS) tissues and cell lines and its expression correlates with prognosis in OS patients. **(A)** Quantitative PCR assays were conducted to measure the *MACC1* mRNA levels in OS tissues and the corresponding adjacent noncancerous tissues (*n* = 30). **(B)** Immunoblot assays were conducted to detect the protein levels of MACC1 in OS tumor tissues and the corresponding adjacent noncancerous tissues. **(C)** Immunoblot assays were conducted to measure the protein levels of MACC1 in the U-2OS, MG-63, Saos-2, and HOS osteosarcoma cell lines, and normal human umbilical vein endothelial cells (HUVECs). **(D)** Immunohistochemistry was performed to measure the protein levels of MACC1 in human OS tissues and the corresponding adjacent noncancerous tissues; representative images are shown (×100 and ×400 magnification, respectively). **(E)** Kaplan–Meyer survival analysis showed the correlation between MACC1 expression in tumor tissues and overall survival or disease-free survival rates.

To assess how MACC1 affects OS progression, we first measured the level of *MACC1* mRNA expression in human OS tissues and the corresponding adjacent noncancerous tissues. Using qPCR, we found that the mRNA levels of *MACC1* were higher in human OS tissue than in adjacent normal tissue (Figure 1A), and this aberrant expression was further confirmed by western blot assays (Figure 1B).

We then investigated the protein expression of MACC1 in 4 OS cell lines (U-2OS, MG-63, Saos-2, and HOS), as well as in the normal osteoblast HCO cell line. In agreement with the results for human OS tissue, immunoblot assays showed that MACC1 protein expression was markedly upregulated in OS cells when compared with that in normal osteoblasts (Figure 1C).

We also performed IHC assays to measure the MACC1 protein expression levels in human OS tissues (Figure 1D), and found that MACC1 was also highly expressed in human OS tissues at the protein level. Based on these results, we further investigated whether the high expression levels of MACC1 were correlated with clinicopathological features of OS. Patients were divided into groups of high or low MACC1 expression based on the staining scores. As shown in Table 2, no significant correlation was detected between MACC1 expression and several of the clinicopathological features, including patient gender (*p* = 0.120), age (*p* = 0.715), and primary site. Importantly, however, high MACC1 expression was correlated with high microvessel density (*p* = 0.020) and tumor volume (*p* = 0.002).

**TABLE 2:** The relationship between MACC1 expression and clinicopathological characteristics.

Clinicopathological characteristic		mRNA expression		*P*-value	Protein expression		*P*-value
		Negative	Positive		Negative	Positive	
Gender	Male	69	76	0.373	74	71	0.120
	Female	42	36		48	30	
Age (years)		2513.8	24.3 ± 14.9	0.739	24.313.9	2514.9	0.715
Primary site	Limbs	83	92	0.181	93	82	0.370
	Others	28	20		29	19	
Tumor staging	I	9	4	0.302	10	3	0.252
	II	93	96		101	88	
	III	9	12		11	10	
Tumor volume	<200 cm^2^	74	57	0.017*	83	48	0.002**
	>200 cm^2^	37	55		39	53	
MVD		27.45.4	29.3 ± 7.1	0.029*	27.45.7	29.46.9	0.020*

We additionally analyzed the difference in survival rate between the high and low MACC1 expression groups. Kaplan–Meyer survival analysis indicated that the median follow-up for 223 OS cases was 38.2 months. As shown in Figure 1E, patients whose samples exhibited low levels of MACC1 expression had substantially improved overall survival (left) and disease-free survival (right) than those whose samples showed high MACC1 protein expression (*p* = 0.030 and *p* = 0.018, respectively), suggesting that a correlation exists between MACC1 expression and prognosis in OS patients.

### MACC1 Promotes the Proliferation of OS Cells *in vitro*

**FIGURE 2:**
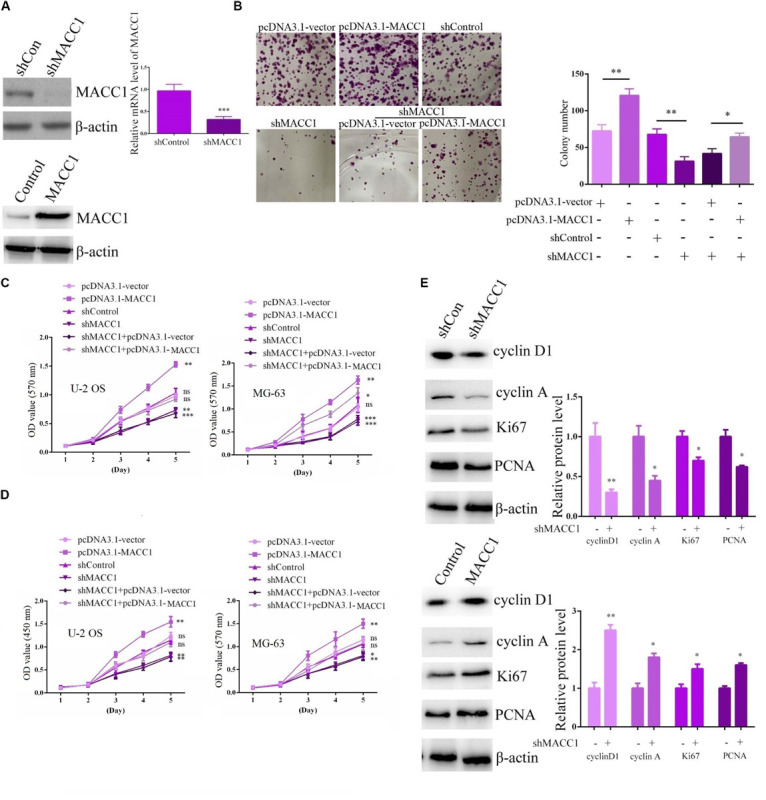
MACC1 promotes the proliferation of osteosarcoma cells *in vitro*. **(A)** Immunoblot assays and qPCR were performed to measure the protein expression of MACC1 in osteosarcoma (OS) cells following transfection with the indicated plasmids. **(B)** Colony formation assays were performed to evaluate the difference in proliferative ability between OS cells in the indicated groups. **(C)** MTT assays were performed to detect the proliferation level in cells transfected with the indicated plasmids. **(D)** CCK-8 assays were performed to detect the proliferation level in cells transfected with the indicated plasmids. **(E)** Immunoblot assays were performed to analyze the expression of Ki-67, PCNA, cyclin D2, and cyclin A in OS cells transfected with the indicated plasmids. Quantification of relative protein level were displayed. The results are presented as means ± SEM; ^∗^*p* < 0.05, ^∗∗^*p* < 0.01, and ^∗∗∗^*p* < 0.001.

To investigate the possible effects of MACC1 on OS progression *in vitro*, we used shRNA plasmids targeting MACC1 and a MACC1 overexpression plasmid (pCDNA3.1-MACC1) to promote changes in MACC1 expression in U-2OS and MG-63 OS cells. The effective silencing of MACC1 expression in OS cells was confirmed by immunoblot assays (Figure 2A), and we also confirmed that MACC1 expression was markedly upregulated in cells transfected with the pcDNA3.1-MACC1 plasmid (Figure 2A).

We then assessed the effects of MACC1 on the proliferative ability of OS cells through colony formation assays. Importantly, we found that MACC1 depletion led to a substantial decrease in the proliferative ability of U-2OS cells, whereas MACC1 overexpression greatly increased the number of viable colonies (Figure 2B). Furthermore, MACC1 overexpression in MACC1-depleted U-2OS cells rescued the inhibition of cell proliferation resulting from MACC1 depletion, showing that MACC1 has a role in the regulation of OS cell proliferation (Figure 2B). We further clarified whether MACC1 has a role in OS cell proliferation using MTT and CCK-8 assays. Consistent with the colony formation assay data, MTT assays showed that depletion of MACC1 led to a decrease in the OD_570_ value, whereas overexpression of MACC1 significantly increased the OD value in both U-2OS and MG-63 cells (Figure 2C). Notably, MACC1 overexpression also rescued the reduced OD value resulting from MACC1 depletion (Figure 2C). CCK-8 assays also showed a similar change in the OD_450_ value following the transfection of U-2OS and MG-63 cells with the indicated plasmids (Figure 2D).

Ki-67 and PCNA are markers of cell proliferation, and high expression of these markers is indicative of induced cell proliferation. Immunoblot analysis indicated that the expression of both Ki-67 and PCNA was downregulated in U-2OS cells after MACC1 knockdown, whereas overexpression of MACC1 had the opposite effect (Figure 2E). The expression of cyclin D1 and cyclin A in U-2OS exhibited similar expression patterns. (Figure 2E). Collectively, these data confirmed that MACC1 has a critical role in the regulation of OS cell proliferation.

### MACC1 Promotes Endothelial Cell Angiogenesis via the Regulation of Microtubule Stability

As the clinicopathological data indicated that MACC1 expression was clearly correlated with microvessel density, MVD, in the tumors of OS patients, we assessed the effects of MACC1 on endothelial cell angiogenesis *in vitro*.

**FIGURE 3:**
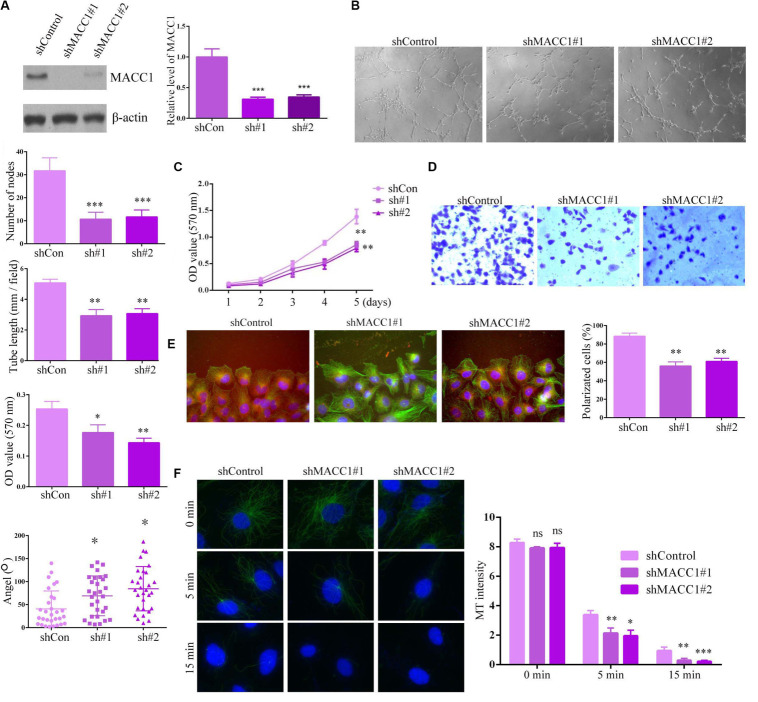
MACC1 promotes endothelial cell angiogenesis *in vitro.* **(A)** Immunoblot assays and QPCR were performed to evaluate the protein expression of MACC1 in endothelial cells following transfection with the indicated plasmids. **(B)** Human umbilical vein endothelial cells (HUVECs) transfected with the indicated shRNA plasmids were plated onto Matrigel to induce tube formation, and images were obtained 3 h later. The degree of tube formation was quantified by measuring the total tube length and number of nodes. **(C)** Representative images of a Transwell migration assay for HUVECs transfected with the indicated shRNA plasmids, and quantification of migrated cells. **(D)** Representative images of wound healing in HUVECs transfected with shRNA plasmids at the indicated times, and quantification of the extent of wound closure. **(E)** Immunofluorescence microscopy images of HUVECs transfected with shRNA plasmids, followed by staining with DAPI, and anti-α-tubulin and anti-γ-tubulin antibodies. Scheme for the measurement of polarization angle. Polarized cells and polarization angles were quantified. **(F)** HUVECs were transfected with the indicated plasmids, incubated on ice to depolymerize the microtubules (MTs), followed by quantification of MT intensity. Representative immunofluorescence images are shown. Results are presented as means ± SEM; ^∗^*p* < 0.05, ^∗∗^*p* < 0.01, and ^∗∗∗^*p* < 0.001.

We transfected two shRNA plasmids targeting MACC1 into HUVECs to deplete MACC1 expression, and confirmed the effective silencing of MACC1 in HUVECs by immunoblot assays (Figure 3A). To investigate the possible effects of MACC1 on angiogenesis, we performed tube formation assays. Interestingly, we found that MACC1 depletion markedly impaired tube formation, suggestive of impaired angiogenesis (Figure 3B). As endothelial cell proliferation and migration are key processes involved in angiogenesis, we further investigated whether depletion of MACC1 would affect endothelial cell proliferation and migration. As expected, MTT assays confirmed that MACC1 depletion suppressed the proliferative ability of HUVECs (Figure 3C). Transwell assays also showed that MACC1 depletion markedly suppressed the migration of HUVECs (Figure 3D). Since polarization is a requisite for cell migration, we analyzed the effect of IFT88 on cell polarization by measuring the relative localization of the centrosomes and nuclei of cells at the edge of the wound. Interestingly, we found that most control cells near the wound exhibited a polarization angle of less than 60°, whereas MACC1 depletion led to an increase in the angle of polarization, as well as impaired cell polarization (Figure 3E).

As all the observed effects of MACC1 on endothelial cell proliferation, migration, and polarization were related to microtubule dynamics and stability, we assessed how MACC1 depletion would affect the microtubules. Interestingly, we found that MACC1 depletion significantly promoted microtubule depolymerization on ice after 5 min and 15 min, suggesting that MACC1 is critical for microtubule stability (Figure 3F). Together, these data confirmed that MACC1 modulates angiogenic processes in a microtubule-dependent manner.

### MACC1 Mediated the Proliferation of High-c-Met Expressed Osteosarcoma Cells, but Not That of HUVECs, via the HGF/c-Met Pathway

**FIGURE 4:**
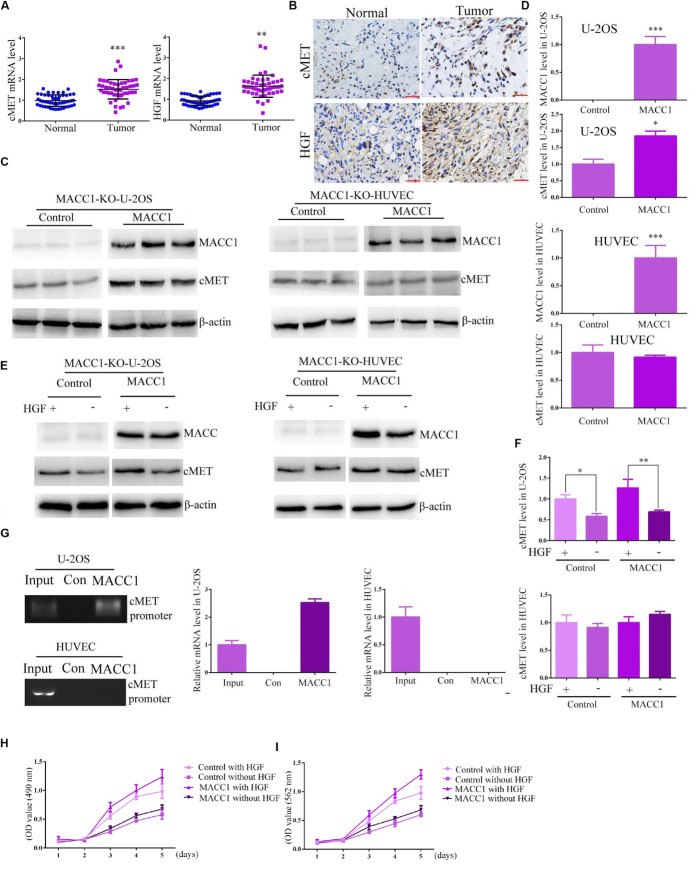
MACC1 differentially regulates HGF/c-Met signaling in osteosarcoma and human umbilical vein endothelial cells (HUVECs). **(A)** The mRNA levels of c-Met (left) and HGF (right) in tumor tissues and the corresponding adjacent noncancerous tissues collected in our hospital (*n* = 30). **(B)** Representative immunohistochemistry images of c-Met and HGF expression. The expression level was compared between normal and tumor tissues. **(C)** MACC1-knockout U-2OS cells and HUVECs were transfected with pcDNA3.1-MACC1 or pcDNA3.1-empty vector plasmids, and the expression levels of MACC1 and c-Met were measured through immunoblot assays, followed by quantification of relative protein levels **(D)**. **(E)** HGF induced the expression of c-Met in U-2OS cells, but not in HUVECs. MACC1-knockout U-2OS cells and HUVECs were transfected with pcDNA3.1-MACC1 or pcDNA3.1-vector plasmids, and then treated with 100 ng of HGF for 24 h. The expression levels of MACC1 and c-Met were measured through immunoblot assays, followed by quantification of relative protein levels **(F)**. **(G)** ChIP assays were performed to investigate whether MACC1 could bind to the promoter region of c-Met in U-2OS cells and HUVECs. A visible band was observed in U-2OS cells, but not HUVECs, following MACC1 plasmid transfection. Relative ratios of IP DNA to input DNA were determined by ChIP-PCR. **(H)** CCK-8 assays were performed upon the indicated treatment in U-2OS cells. **(I)** MTT assays were performed upon the indicated treatment in U-2OS cells. Results are presented as means ± SEM; ^∗∗^*p* < 0.01 and ^∗∗∗^*p* < 0.001.

We then investigated the possible mechanisms underlying the MACC1-mediated promotion of OS cell proliferation. As previous studies have suggested that the possible effects of MACC1 on cancer cell proliferation are mediated *via* the HGF/c-Met signaling pathway, we explored whether the MACC1-induced effects on OS were mediated *via* the HGF/c-Met pathway. Quantitative PCR analysis indicated that the mRNA levels of HGF and c-Met were increased in human OS tissues when compared with those of normal tissues (Figure 4A), and similar results were obtained using IHC assays (Figure 4B). We further used qPCR to detect HGF expression and MACC1 expression in 30 OS tissues from the patients. We analyzed the expression and found that in OS tissues with high MACC1 expression, the expression of HGF was relatively low, which confirmed the negative correlation between MACC1 and HGF mRNA levels, suggesting that MACC1 could negatively regulate the expression of HGF (Supplementary Figure S1). This suggested that both HGF and c-Met may be involved in OS progression.

We used MACC1-knockout U-2OS cells and MACC1-knockout HUVECs that do not express endogenous MACC1. Following pcDNA3.1-empty vector or pcDNA3.1-MACC1 plasmid transfection into MACC1-knockout U-2OS cells and HUVECs, a significant increase in the protein expression of c-Met was observed in U-2OS cells, but not in HUVECs (Figures 4C,D). HGF is widely accepted to be a ligand for the c-Met receptor, and binding of HGF to c-Met can promote the translocation of MACC1 from the cytoplasm into the nucleus. Therefore, we compared the HGF-induced expression of c-Met in MACC1-knockout U-2OS cells and HUVECs. As shown in Figure 4E, U-2OS cells transfected with pcDNA3.1-MACC1 expressed higher c-Met levels than cells transfected with the pcDNA3.1-empty vector plasmid. However, transfection of pcDNA3.1-MACC1 into HUVECs elicited no significant effect on HGF-induced c-Met expression (Figures 4E,F). This indicated that MACC1 regulates the proliferation of OS cells, but not that of HUVECs, *via* the HGF/c-Met signaling pathway.

To further confirm our conclusion, we tried to screen an OS cell line with relative low c-Met expression. According to the results of Immunoblot assays, we found the expression of c-Met in Saos-2 cells were obviously lower than other types of OS cells (Supplementary Figure S2). Therefore we assessed whether MACC1 regulated the proliferation of OS cells via a similar pathway. We then used MACC1 shRNA plasmids to deplete the expression of MACC1 in Saos-2 cells, which was confirmed by Immunoblot and q-PCR assays (Supplementary Figures S3A,B). Notably, we found a relative modest effect of MACC1 on the proliferation of Saos-2 cells, confirmed by MTT and CCK-8 assays (Supplementary Figure S3C,D). Overexpression, depletion or rescue assays all confirmed the modest effect of MACC1 on Saos-2 cell proliferation (Supplementary Figure S3C,D). We therefore guessed that c-Met expression was play a critial role in MACC1 mediated OS progression.

We then constructed and used MACC1-knockout Saos-2 cells that do not express endogenous MACC1. However, we found following pcDNA3.1-empty vector or pcDNA3.1-MACC1 plasmid transfection into MACC1-knockout Saos-2 cells, a modest change in the protein expression of c-Met was noticed in Saos-2 cells (Supplementary Figure S4), additionally, Saos-2 cells transfected with pcDNA3.1-MACC1 expressed a similar c-Met levels compared to cells transfected with the pcDNA3.1-empty vector plasmid (Supplementary Figure S5). Therefore, we thought MACC1 mediated the proliferation of high c-Met expressed OS cells.

We then performed chromatin immunoprecipitation (ChIP) assays to explore whether MACC1 could bind to the *MET* promoter in U-2OS cells and HUVECs. As expected, we found that MACC1 could bind to the *MET* promoter in U-2OS cells, and a visible band for the *MET* promoter was found for U-2OS cells transfected with pcDNA3.1-MACC1 (Figure 4G). However, no similar results were found in HUVECs (Figure 4G). Similarly, we further verified MACC1 IP DNA was enriched in the cMET promoter in U-2OS cells but not in HUVECs by ChIP-PCR results. We further performed CCK-8 assays and MTT assays upon the same treatment in U-2OS cells, and found MACC1 contributed to the proliferation of OS cells via HGF-cMET axis (Figures 4H,I). We therefore thought MACC1 mediated the proliferation of OS cells, but not that of HUVECs, via the HGF/c-Met pathway.

### MACC1 Depletion Suppressed the Tumor Growth of OS Cell Xenografts in Mice

**FIGURE 5:**
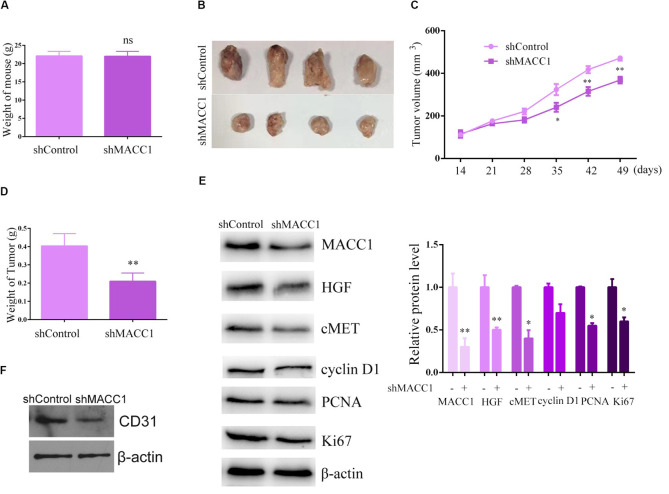
MACC1 depletion inhibits osteosarcoma (OS) cell xenograft tumor growth in mice. U-2OS cells infected with control or MACC1 shRNA lentiviral vectors were subcutaneously implanted into nude mice. After 2 weeks, the tumors were isolated and tumor volume was examined every 7 days (*n* = 6 in each group). **(A)** Mouse weight was compared between control and MACC1 depletion groups. **(B)** Representative tumor images are shown; a tumor growth curve was calculated and analyzed according to the average volume of 6 tumors in each group. **(C)** Tumor volume was compared between control and MACC1 depletion groups. **(D)** Tumor weight was compared between control and MACC1 depletion groups. **(E)** Immunoblot assays were performed to assess the expression levels of the indicated proteins in control or MACC1-depleted tumor tissues isolated from nude mice. **(F)** Immunoblot assays were performed to assess the expression levels of the indicated proteins in control or MACC1-depleted tumor tissues isolated from nude mice. Quantification of relative protein level were displayed. Results are presented as means ± SEM; ^∗^*p* < 0.05, ^∗∗^*p* < 0.01, and ^∗∗∗^*p* < 0.001.

The above results showed that downregulation of MACC1 inhibited OS cell proliferation, and suppressed endothelial cell angiogenesis *in vitro*. To explore the potential role of MACC1 in OS tumor growth *in vivo*, U-2OS cells were infected with control or MACC1-targeted shRNA lentiviral particles and subcutaneously injected into nude mice. Xenograft tumor formation began after 2 weeks. First, we measured the weight of mice in the control and MACC1-depleted groups, and found no difference between the weights of the two groups (Figure 5A). The tumors were subsequently isolated from the mice and imaged, while tumor volume was measured every 7 days. Images of representative tumors are shown in Figure 5B, and the calculated growth curve is shown in Figure 5C. Interestingly, the volume of the tumors isolated from the MACC1-depleted group was significantly smaller than that of the control group (Figure 5D). Consistent with this result, tumor weight was also markedly decreased by MACC1 depletion.

Immunoblot assays confirmed the effective downregulation of MACC1 expression in tumor tissues from the MACC1-depleted groups (Figure 5E). We also found that the expression of Ki-67, cyclin D2, and cyclin A was markedly reduced in tumor tissues from MACC1-depleted groups than in those from the controls (Figure 5E). Importantly, our data also demonstrated that the expression of c-Met and HGF was reduced in MACC1-depleted tumor tissues (Figure 5E), further confirming our previous findings. Additionally, we noticed the expression of CD31 in tumor tissues from MACC1 depletion group was obviously decreased, compared to the control group, suggesting the decrease number of endothelial cells and confirmed our *in vitro* data (Figure 5F). Combined, the *in vivo* results showed that MACC1 is involved in the regulation of OS tumor growth *via* the MACC1/HGF/c-Met signaling axis and the regulation of angiogenesis.

## Discussion

**FIGURE 6:**
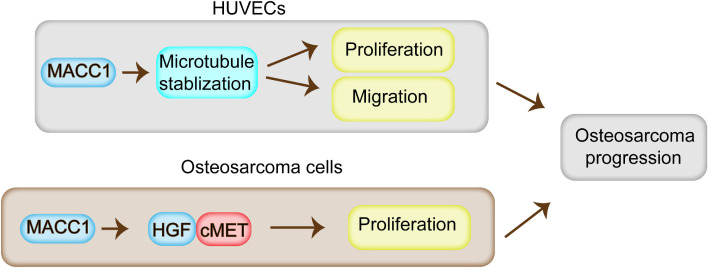
Model for the regulatory role of MACC1 in osteosarcoma progression. MACC1 promotes cell proliferation through the HGF/c-Met pathway, and contributes to the proliferation, migration, and angiogenesis of endothelial cells *via* the regulation of microtubule stability, which further promotes osteosarcoma progression.

In this study, we found that MACC1 is highly expressed in human OS tissues. We further found that correlations existed between MACC1 expression, patient prognosis, and clinical characteristics. Through *in vitro* and *in vivo* assays, we demonstrated that MACC1 activity increases the proliferative capacity of OS cells *via* the HGF/c-Met pathway, and stimulates angiogenesis through the regulation of microtubule stability (Figure 6). Together, our findings confirmed that MACC1 is a potential therapeutic target for the treatment of OS.

Osteosarcoma is a malignancy derived from the skeletal system that has no clearly identified prognostic factors. Importantly, the incidence of OS is increasing annually, and the overall five-year survival rate is still low owing to the high heterogeneity and metastasis of advanced OS, as well as the lack of effective treatment (Anderson, 2016). In recent years, targeted therapy has led to a significant improvement in the prognosis of OS patients (Endo-Munoz et al., 2019). For highly metastatic tumors, while liposomal drug delivery system has achieved good results (Yang et al., 2020), targeted therapy for protein targets is more common. Notably, several molecular targets, such as GSK3B and ALCAM, have been identified, and some targeted therapies are in clinical trials (Nishimura et al., 2016). However, to further improve the prognosis of OS patients, suitable therapeutic targets are still needed. Importantly, we found that MACC1 is highly expressed in human OS tissues and cell lines. Our data further confirmed that MACC1 expression was correlated with the prognosis and several clinicopathological features of OS patients, suggesting that MACC1 may be a promising therapeutic target in OS.

Metastasis-associated in colon cancer 1 is known to be involved in the progression of more than 10 solid tumors and hematologic tumors (Stein, 2013). Overexpression of MACC1 paracrine signaling in carcinoma-associated fibroblasts has been shown to contribute to invasion by lung adenocarcinoma cells (Li et al., 2019). Moreover, MACC1 has also been reported to increase the proliferative and apoptotic capacity of lung adenocarcinoma cells by acting on the β-catenin pathway, suggesting that MACC1 might regulate tumor progression through various signaling pathways (Guo et al., 2018). In addition, MACC1 was also shown to regulate the proliferation, apoptosis, migration, and invasion of squamous cell carcinoma cells *via* the induction of autophagy (Wu et al., 2017). Through colony formation, MTT, and CCK-8 assays, we found that MACC1 enhances the proliferative capacity of OS cells *in vitro*. Our data further confirmed that the expression of Ki-67 and PCNA, two cell markers of proliferation, was correlated with MACC1 expression. Mechanistically, we found that MACC1 promotes the proliferation of OS cells *via* the MACC1/c-Met/HGF axis, both *in vitro* and in a xenograft OS mouse model. These findings, together with those of other studies, confirmed that MACC1 has a significant role in cancer progression. The current priority is to develop MACC1 inhibitors to test their potential antitumor effects.

The HGF/c-Met signaling pathway affects multiple cellular processes during cancer progression, including cell proliferation, migration, invasion, apoptosis, and drug-resistance (Stein et al., 2009; Sheng et al., 2014; Sueta et al., 2015; Li et al., 2018). In this study, we identified that MACC1 modulates OS progression *via* the MACC1/HGF/c-Met axis. Several studies have previously shown that a correlation exists between MACC1 and the HGF/c-Met pathway. Metastasis-associated in colon cancer 1 was shown to play a differential role in breast and colorectal cancer *via* HGF/c-Met signaling (Sueta et al., 2015). Similarly, MACC1 and HGF levels were correlated with the survival rates of gastric cancer patients (Guo et al., 2013; Dong et al., 2018), while a different study also demonstrated that MACC1 promotes the progression of epithelial ovarian cancer *via* the HGF/c-Met signaling pathway (Sheng et al., 2014). These studies demonstrated the important role of the HGF/c-Met pathway on cancer progression, and suggested that MACC1 may be a potential molecular target linked to this pathway.

Our conclusion confirmed MACC1 regulated OS through HGF-cMet axis, however, in OS cells with low or no c-Met expression, such as in Saos-2 cells, the effects of MACC1 on cell proliferation had been limited. When the background expression of c-Met was low in OS tissues, MACC1 had the limited capacity to induce c-Met expression. Additionally, there was also no significant change in c-Met expression after HGF treatment in control or MACC1 overexpression cells. It is important to note that we had detected the c-Met expression in tumor samples, and the results showed that the c-Met in tumor tissues was significantly higher expression compared with adjacent tissues, and the expression level of c-Met in vast majority of tumor samples were higher than that in adjacent tissues, which was consistent with previous studies. Therefore, we thought MACC1 regulated OS proliferation through HGF-cMet axis.

Notably, the low expression of c-Met in tumor tissues did not affect the regulation of MACC1 on endothelial cell angiogenesis, a critical process for cancer progression. Therefore, MACC1 also could promote the progression of OS with relative low c-Met expression, through promoting angiogenesis.

We also noticed that MACC1 enhanced epithelial cell angiogenesis in a microtubule-dependent manner (Peng et al., 2019). Angiogenesis comprises endothelial cell migration, proliferation, and basement membrane reconstruction, as well as other key processes. Through MTT and Transwell assays, we found that depletion of MACC1 suppressed the proliferation and migration of endothelial cells. Additionally, as cell polarization is a prerequisite for migration, we assessed whether MACC1 also regulates cell polarization. As expected, we found that MACC1 depletion impaired the polarization ability of endothelial cells and significantly stimulated microtubule depolymerization on ice. These results provide strong evidence that MACC1 promotes angiogenesis through mediating microtubule stability. Previous studies have also suggested that MACC1 could modulate cytoskeletal dynamics through its ZU5-UPA-DD domain (Cao et al., 2019); However, the precise molecular mechanisms involved require further study. A different study demonstrated that MACC1 promotes angiogenesis in cholangiocarcinoma by upregulating VEGFA expression (Peng et al., 2019). In OS, we observed that MACC1 depletion had a modest effect on VEGFA expression levels (data not shown), suggestive of a differential regulatory mechanism for MCCA1 between OS and cholangiocarcinoma. We also detected that MACC1 moderately affected microtubule stability in OS cells, suggesting that MACC1 may have specific effects on endothelial cells.

## Data Availability Statement

All datasets generated for this study are included in the article/Supplementary Material.

## Ethics Statement

The studies involving human participants were reviewed and approved by the Ethics Committee of the First Affiliated Hospital of Zhengzhou University. The patients/participants provided their written informed consent to participate in this study. The animal study was reviewed and approved by the Ethics Committee of the First Affiliated Hospital of Zhengzhou University. Written informed consent was obtained from the individual(s) for the publication of any potentially identifiable images or data included in this article.

## Author Contributions

JW, YX, and YQZ performed all the experiments, and designed and conducted the animal experiments. YaZ, XL, YiZ, YL, TL, and LL analyzed the data. JW, JZL, and JPL conceived and participated in the design of the study. JW wrote the manuscript. All authors read and approved the final version of the manuscript.

## Conflict of Interest

The authors declare that the research was conducted in the absence of any commercial or financial relationships that could be construed as a potential conflict of interest.
