# Supplementary material for: MACC1 Contributes to the Development of Osteosarcoma Through Regulation of the HGF/c-Met Pathway and Microtubule Stability
Source: Front Cell Dev Biol. 2020 Dec 23;8:825. doi: 10.3389/fcell.2020.00825 (PMC7793648; doi:10.3389/fcell.2020.00825)
Supplement: Supplementary file 1 [file Image_1.pdf]

Supplementary Material

1 Supplementary Figure

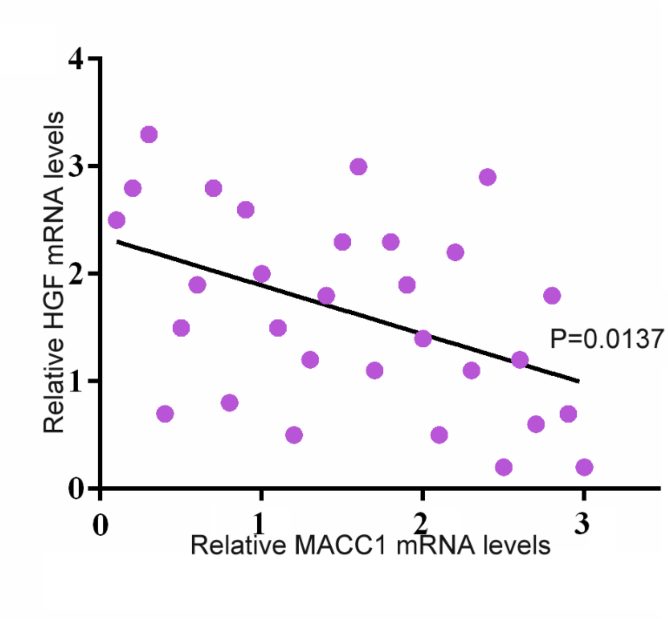

Supplementary Figure S1. The negative correlation between MACC1 and HGF mRNA levels in human osteosarcoma tissues. qPCR assays were performed to detect HGF expression and MACC1 expression in 30 osteosarcoma tissues from the patients.

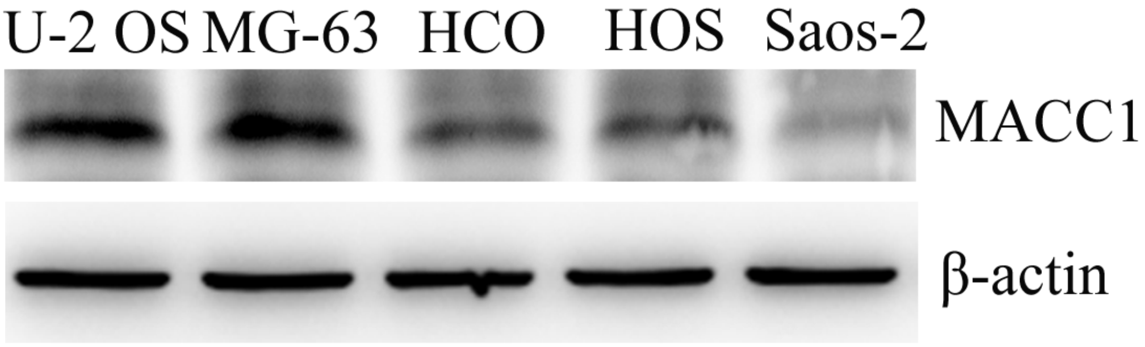

Supplementary Figure S2. The expression of c-Met in different types of OS cells. Immunoblot assays were performed to detect the protein expression of c-Met in the indicated types of cells.

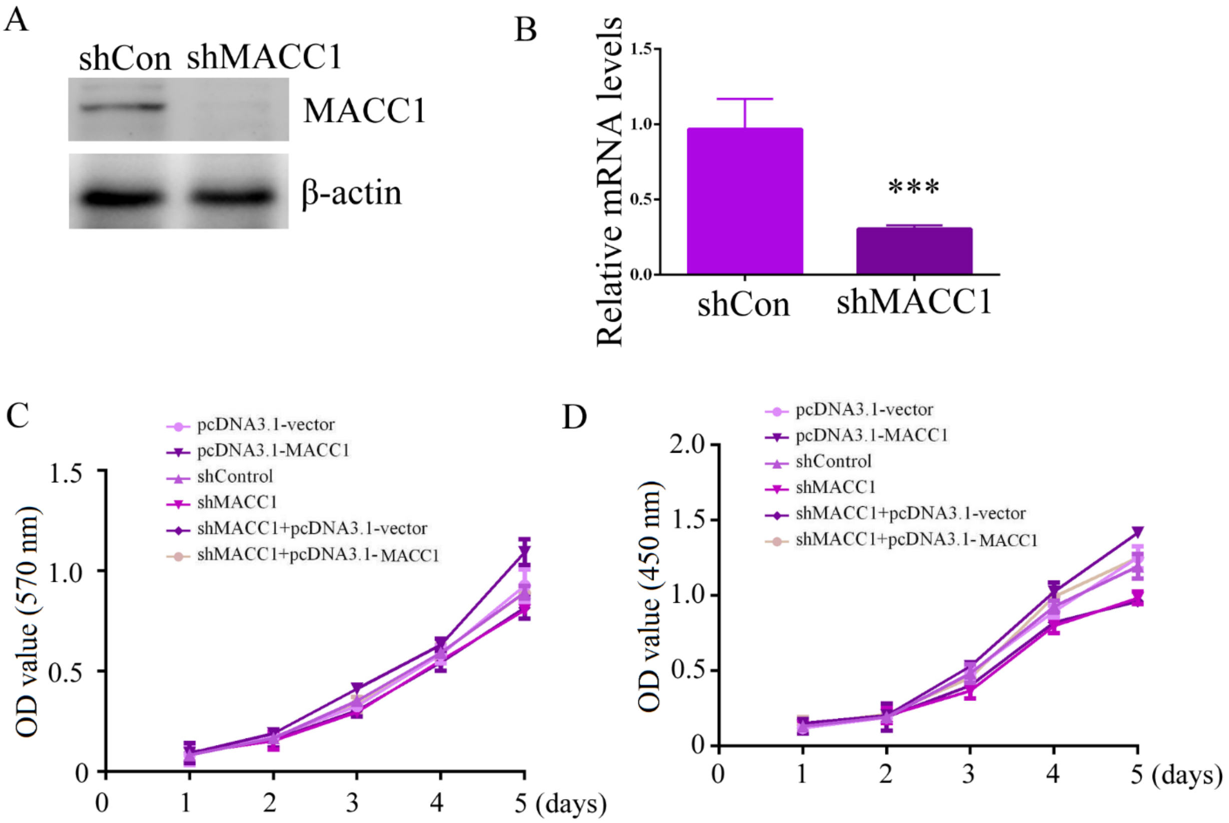

Supplementary Figure S3. MACC1 had relative modest effects on the proliferation of low-c-Met expression OS cells. (A) Immunoblot assays and qPCR were performed to measure the protein expression of MACC1 in Saos-2 cells following transfection with the indicated plasmids. (B). MTT assays were performed to detect the proliferation level in Saos-2 cells transfected with the indicated plasmids. (C,D) MTT (C) and CCK-8 (D) assays were performed to detect the proliferation level in Saos-2 cells transfected with the indicated plasmids. Results are presented as means ± SEM.

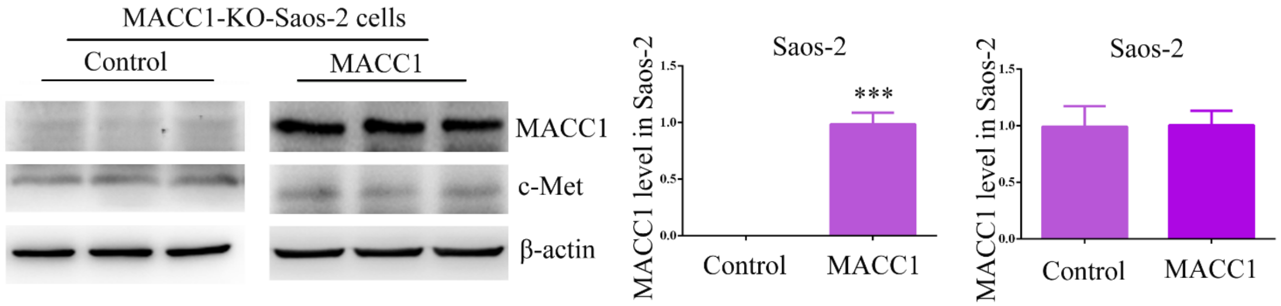

Supplementary Figure S4. MACC1-knockout Saos-2 cells were transfected with pcDNA3.1-MACC1 or pcDNA3.1-empty vector plasmids, and the expression levels of MACC1 and c-Met were measured through immunoblot assays, followed by quantification of relative protein levels.

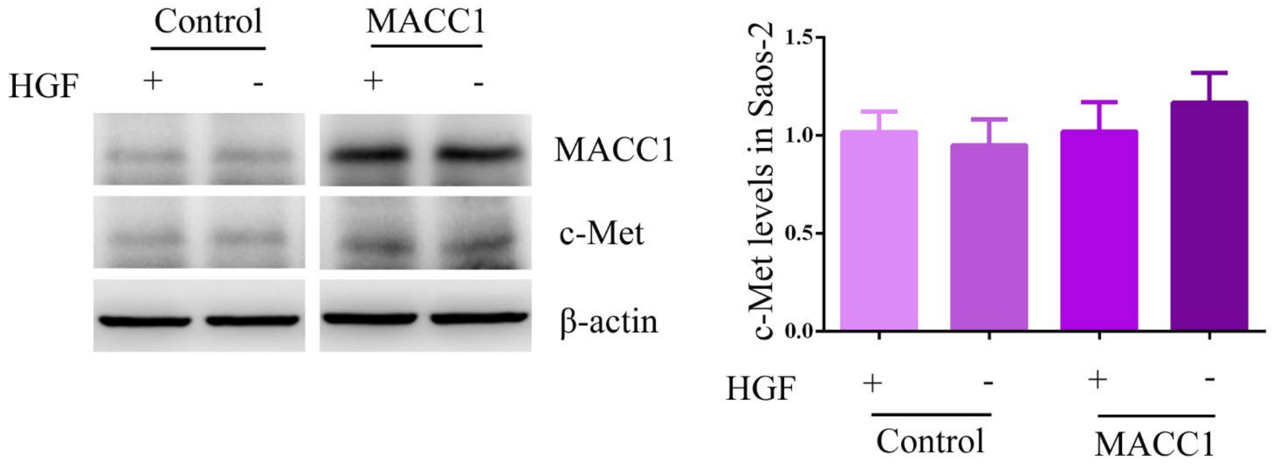

Supplementary Figure S5. HGF treatment had modest effects on the expression of c-Met in Saos-2 cells. MACC1-knockout Saos-2 cells were transfected with pcDNA3.1-MACC1 or pcDNA3.1-vector plasmids, and then treated with 100 ng of HGF for 24 h. The expression levels of MACC1 and c-Met were measured through immunoblot assays, followed by quantification of relative protein levels. Results are presented as means ± SEM.
